# The Atypical Antipsychotic Lurasidone Affects Brain but Not Liver Cytochrome P450 2D (CYP2D) Activity. A Comparison with Other Novel Neuroleptics and Significance for Drug Treatment of Schizophrenia

**DOI:** 10.3390/cells11213513

**Published:** 2022-11-06

**Authors:** Przemysław J. Danek, Władysława A. Daniel

**Affiliations:** Department of Pharmacokinetics and Drug Metabolism, Maj Institute of Pharmacology, Polish Academy of Sciences, Smętna 12, 31-343 Kraków, Poland

**Keywords:** cytochrome P450 2D (CYP2D), brain, liver, lurasidone, enzyme expression and activity

## Abstract

The aim of this work was to study the effect of prolonged lurasidone administration on the cytochrome 2D (CYP2D) expression and activity in the rat liver and selected brain structures involved in the therapeutic or side effects of this neuroleptic. Male Wistar rats received lurasidone (1 mg/kg ip.) for two weeks. The activity of CYP2D was measured in brain and liver microsomes as the rate of bufuralol 1′-hydroxylation. The CYP2D protein level was determined in microsomes by Western blot analysis. The CYP2D gene expression was estimated in liver tissue by a qRT-PCR method. Lurasidone decreased the activity and protein level of CYP2D in the frontal cortex but increased them in the striatum, nucleus accumbens, brain stem, substantia nigra, and the remainder of the brain. The neuroleptic did not affect CYP2D in the hippocampus, hypothalamus, and cerebellum. In the liver, lurasidone did not affect the CYP2D activity and protein level, though it enhanced the mRNA of *CYP2D1* without affecting that of *CYP2D2*, *CYP2D3*, *CYP2D4*, and *CYP2D5*. In conclusion, lurasidone regulates brain (but not liver) CYP2D activity/protein level in a region-dependent manner, which is similar to that of other atypical neuroleptics (iloperidone and asenapine) as concerns the frontal cortex (down-regulation) and nigrostriatal pathway (up-regulation) and may be of pharmacological significance. However, further molecular studies with selective receptor agonists are necessary to find out which individual monoaminergic receptors/signaling pathways are involved in the regulation of the rat CYP2D4 and human CYP2D6 enzyme in particular brain structures.

## 1. Introduction

Cytochrome P450 (CYP) enzymes are a family of heme-containing monooxygenases responsible for metabolizing xenobiotic and endogenous compounds. CYP2D is an important enzyme, as it metabolizes approximately 20% of clinically used drugs, including CNS and cardiovascular system therapeutics, neurotoxins, and endogenous compounds [1,2].

CYP2D enzymes have been detected in various species. In humans, only one enzyme (CYP2D6) is expressed in various tissues, while rats have six enzymes (CYP2D1, 2, 3, 4, 5, and 18) with unique substrate specificity, expression, metabolism, and inhibition properties [3,4]. In the rat, the enzymes CYP2D1 and CYP2D2 are the most abundant CYP2D isoforms in the liver, while CYP2D4 is mostly expressed in the brain [5]. However, rat CYP2D enzymes collectively are a useful model of human CYP2D due to their relatively high amino acid sequence identity (>70%) and the ability to perform metabolic reactions in vitro similar to human CYP2D6 [6].

CYPs are expressed in the brain of numerous animals (mice, rats, monkeys, dogs, and humans), where they may contribute to the local metabolism of exogenous and endogenous compounds and to neuroprotection [7]. Moreover, brain CYP2Ds were shown to contribute to the local metabolism of neurosteroids and to the biosynthesis of dopamine and serotonin [2]. Brain CYPs differ from their hepatic CYP counterparts in their sensitivity to xenobiotic inducers in the isoform profile, brain-region distribution, and mechanism of specific action [8]. In the liver, the CYP2D enzymes are generally regulated by genetic factors and are considered constitutively expressed. Brain CYP2D activity can be modified by both genetic mechanisms and by exposure to xenobiotic inducers [2,8,9].

Lurasidone has been approved by FDA for the treatment of schizophrenia and bipolar depression. This atypical antipsychotic, based on its pharmacodynamic profile, exhibits both antipsychotic and antidepressant action [10]. This atypical neuroleptic drug, like other second-generation atypical neuroleptics, has a high affinity for 5-HT_2A_ and D_2_ receptors and shows antipsychotic efficacy and ability to alleviate some negative symptoms [11]. It also has a strong antagonistic action at 5-HT_7_ receptors and noradrenergic α_2C_ receptors and is a partial agonist of 5-HT_1A_. Its blocking ability toward noradrenergic α_1_ and α_2A_, dopamine D_3_ and D_4_, and serotonin 5-HT_2C_ receptor is weak [12]. Blockade of the D_2_ receptor is responsible for the antipsychotic effect of this drug, while inhibition of 5-HT_2A_ is mainly responsible for its atypical profile. Strong antagonism at 5HT_7_ receptors is related with mood regulation and procognitive effects [13]. Lurasidone is a substrate of the CYP3A enzyme. It is broken down to two main metabolites (ID-20219 and ID-20220), which both have no action at the dopaminergic (D_2_) and serotoninergic (5-HT_1A_, 5HT_2A_, and 5-HT_7_) receptors [14].

Our earlier studies with other atypical drugs, asenapine and iloperidone, showed that chronic neuroleptic treatment produced drug- and brain region-dependent effects on CYP2D protein level and activity in the brain [15,16], some of which were common. Recent in vitro results with pooled human liver microsomes and microsomes from baculovirus-infected insect cells expressing human CYPs (Supersomes) showed a low potency of lurasidone to inhibit the activity of CYP2D6 [17]. Therefore, the main goal of this work was to investigate the effect of lurasidone on the CYP2D expression and activity in the rat liver and selected brain structures involved in the therapeutic or side effects of the neuroleptic.

## 2. Materials and Methods

### 2.1. Animals

All experimental procedures comply with the guidelines of the European regulations for animal experimentation according to the 2010/63/EU Directive and Guide for the Care and Use of Laboratory Animals and with the recommendation of the Local Ethics Commission for Experimentation on Animals at the Maj Institute of Pharmacology, Polish Academy of Sciences, Kraków. Wistar Han rats (males, 3 months old and weighing 270–300 g) from Charles River Laboratories (Sulzfeld, Germany) were used. The rats were maintained in a controlled environment (12:12 light/dark cycle, humidity 50 ± 5%, and temperature 22 ± 2 °C) with typical laboratory food and tap water freely available.

### 2.2. Drugs and Chemicals

Lurasidone hydrochloride was obtained from TargetMol (Boston, MA, USA). For the measurement of CYP2D enzyme activity, the following reagents were used: glucose-6-phosphate, glucose-6-phosphate-dehydrogenase, NADP, RNA-free water, Tween 80, methylcellulose, bufuralol, and 1′-hydroxybufuralol (Sigma, St. Louis, MO, USA). Organic solvents with HPLC purity were from Merck (Darmstadt, Germany). For the analysis of the CYP2D gene expression, the next components were used: Total RNA Mini kit (A&A Biotechnology, Gdynia, Poland), High-Capacity cDNA Reverse Transcription Kit, TaqMan assay, and the TaqMan Gene Expression Master Mix (Life Technologies, Carlsbad, CA, USA). For the determination of the CYP2D protein level, the following reagents were used: the polyclonal antihuman CYP2D6 antibody (FineTest, Wuhan, China), polyclonal rabbit antirat CYP2D4 antibody (University Medical School, Osaka, Japan), goat antirabbit secondary antibodies (Vector Laboratories, Burlingame, CA, USA), mouse antirat β-actin antibody (Sigma, St. Louis, MO, USA), goat antimouse antibody (Jackson ImmunoResearch, West Grove, PA, USA), cDNA-expressed rat CYP2D4 (Bactosomes) (Cypex, Dundee, Scotland, UK), cDNA-expressed human CYP2D6 (Supersomes) (Gentest Corp., Woburn, MA, USA), SignalBoost™ Immunoreaction Enhancer Kit (Millipore, Burlington, MA, USA), Laemmli sample buffer (Bio-Rad, Hercules, CA, USA), and nitrocellulose membranes (Merck, Darmstadt, Germany).

### 2.3. Animal Treatment and Preparation of Brain and Liver Microsomes

Rats (*n* = 15 for each treatment group) received intraperitoneally a pharmacological dose of lurasidone (1 mg/kg ip.) or vehicle (0.5% methylcellulose and 0.2% Tween 80 in sterile water) once daily for two weeks [18,19,20]. Rats were sacrificed by decapitation 24 h after the last dose. Whole livers and brains were removed, and the selected brain structures (in accordance with the Paxinos and Watson atlas [21]) (the cerebellum, brain stem, substantia nigra, hippocampus, frontal cortex, hypothalamus, striatum, nucleus accumbens, and the remainder of the brain) were isolated. All those tissues were immediately frozen in dry ice and then stored at −80 °C until analysis. The differential centrifugation methods [22,23] were used to obtain liver and brain microsomes.

### 2.4. Measurement of the CYP2D Enzyme Activity in Brain and Liver Microsomes

The CYP2D enzyme activity was determined using the CYP2D specific metabolic reaction, i.e., 1′-hydroxylation of bufuralol. Briefly, the incubation system included: 2 mM potassium phosphate buffer (pH = 7.4) and NADPH generating system (1.6 mM NADP, 4 mM MgCl_2_, 5 mM glucose 6-phosphate, and 2.5 U glucose 6-phosphate-dehydrogenase). The protein concentration in microsomes derived from selected brain structures of 1–3 rats (mg of protein/mL: ca. 0.3 for the nucleus accumbens and substantia nigra; 2.8 for the frontal cortex and remainder of the brain; 1.8 for the hippocampus, hypothalamus and the cerebellum; 1.2 for the brain stem and the striatum) or liver microsomes (0.5 mg of protein/mL) was used, as described earlier [15,24]. The appropriate concentrations of bufuralol (brain microsomes—125 µM or liver microsomes—10 µM) were applied in the final volume of 0.4 mL. The incubation time for brain microsomes was 60 min and for liver microsomes was 10 min at a temperature of 37 °C.

In all experiments, the amount of specific metabolic product (1′-hydroxybufuralol formed from bufuralol) was measured by an HPLC method with fluorometric detection [25].

### 2.5. Evaluation of CYP2D Protein in Brain and Liver Microsomes

The CYP2D protein levels in the brain and liver microsomes of control and lurasidone-treated rats were estimated by Western immunoblot analysis, as described previously [15]. The total protein concentration in samples was determined in the microsomes using the Lowry methods [26]. All samples were heated in a Laemmli sample buffer for 5 min at 100 °C. Next, the microsomal proteins (10 μg) were separated using an SDS polyacrylamide gel electrophoresis and transferred onto a nitrocellulose membrane. The blots were probed with the following primary antibodies: rabbit antirat CYP2D4 (1:1000) or rabbit antihuman CYP2D6 (1:2000). In order to dilute the primary and secondary antibodies, the specified solutions from the SignalBoost™ Immunoreaction Enhancer Kit (Merck Millipore, Burlington, MA, USA) were used. As a positive control, we used rat cDNA-expressed CYP2D4 (2.5 µg) or human CYP2D6 (1 µg). The blots were visualized using the Luminescent Image Analyzer LAS-1000 and Image Gauge 3.11 programs (Fuji Film, Tokyo, Japan). The results were normalized to β-actin protein level.

### 2.6. Examination of the Expression of Genes Coding for CYP2D Enzymes in the Liver

The total RNA was extracted from the liver samples using Total RNA Mini kit. RNA quality was determined by chip-based capillary electrophoresis using an Agilent Bioanalyzer 2100 (Agilent, Palo Alto, CA, USA). The quantity of the isolated RNA was verified using a Synergy/HTX multimode reader (BioTek, Winoosk, VT, USA). RNA (1 μg) was reverse transcribed to cDNA using High Capacity cDNA Reverse Transcription Kit. RT-PCR was performed using the Bio-Rad CFX96 PCR system (Bio-Rad, Hercules, CA, USA). TaqMan Gene Expression Assays (Thermo Fisher Scientific, Waltham, MA, USA) were used, including Rn01775090_mH (*CYP2D1*), Rn00562419_m1 *(CYP2D2*), Rn00597330_m1 *(CYP2D3*), Rn00593393_m1 *(CYP2D4*), Rn01790051_s1 *(CYP2D5*), and Rn00667869_m1 (*ACTB)*, which was used for normalization. Gene expression was determined using 2-delta Ct method, as reported previously [27].

### 2.7. Data Analysis

The statistical significance of alterations in enzyme activity, protein level, or gene expression was calculated using Student’s *t*-test compared to the control value (GraphPad Prism 8.0; GraphPad Prism Software Inc., CA, USA). The results of chronic lurasidone treatment are reported as the mean ± S.E.M. The changes were considered as statically significant when *p* < 0.05.

## 3. Results

### 3.1. The Effect of Chronic Lurasidone Treatment on the CYP2D Activity in the Brain and Liver Microsomes of Rats

**Figure 1:**
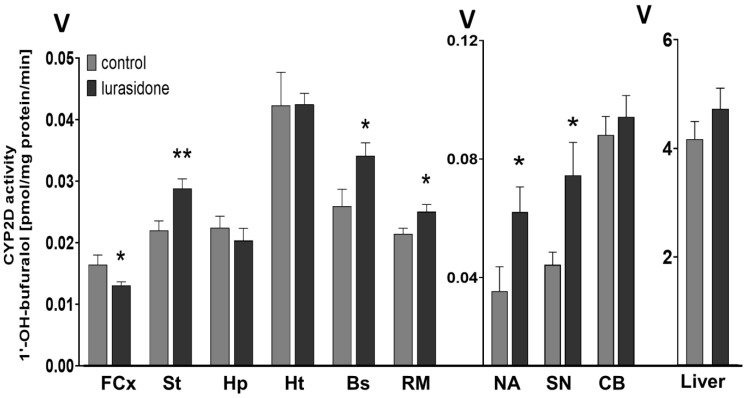
The influence of the two-week treatment with lurasidone on the CYP2D activity measured in microsomes derived from the selected brain structures or liver. The presented values are the means ± S.E.M. of 15 samples (from 15 animals) for the cerebellum, remainder of brain, and the liver; of 7 samples (each sample contained 2 pooled brain structures from 2 animals) for the frontal cortex; brain stem, striatum, and hippocampus; and of 5 samples (each sample contained 3 pooled brain structures from 3 animals) for the hypothalamus, nucleus accumbens, and substantia nigra. Student’s *t*-test: * *p* < 0.05; ** *p* < 0.01 vs. control group. FCx—the frontal cortex, St—the striatum, NA—the nucleus accumbens, Hp—the hippocampus, Ht—the hypothalamus, Bs—the brain stem, SN—the substantia nigra, CB—the cerebellum, and RM—the remainder.

The activity of CYP2D measured as the rate of bufuralol 1′-hydroxylation was significantly changed in different brain regions after prolonged administration of lurasidone (Figure 1). A two-week treatment with lurasidone diminished the activity of CYP2D in the frontal cortex (to 77% of control) but increased the enzyme activity in the striatum (to 131% of the control), nucleus accumbens (to 182% of control), brain stem (to 133% of control), substantia nigra (to 156% of control), and the remainder of the brain (to 115% of control). In contrast, the examined neuroleptic did not affect significantly the CYP2D activity in the hippocampus, hypothalamus, cerebellum, and in the liver (Table 1).

### 3.2. The Effect of Chronic Lurasidone Treatment on the CYP2D Protein Level in Microsomes from the Brain and Liver

**Figure 2:**
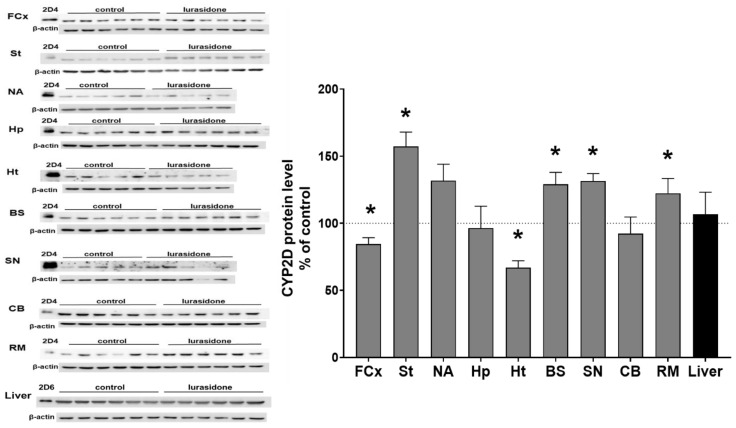
The effect of two-week treatment with lurasidone on the CYP2D protein levels measured in microsomes derived from the selected brain structures or liver. The presented values are the means ± S.E.M. of 15 samples (from 15 animals) for the cerebellum, remainder of brain, and the liver; of 7 samples (each sample contained 2 pooled brain structures from 2 animals) for the frontal cortex, brain stem, striatum, and hippocampus; or of 5 samples (each sample contained 3 pooled brain structured from 3 animals) for the hypothalamus, nucleus accumbens, and substantia nigra. The representative CYP2D protein bands of the Western blot analysis are shown. Brain or liver microsomal protein (10 µg) was subjected to Western blot analysis. cDNA-expressed CYP2D4 protein (Bactosomes) and cDNA-expressed CYP2D6 protein (Supersomes) was used as a positive control. Student’s *t*-test: * *p* < 0.05 vs. control group. FCx—the frontal cortex, St—the striatum, NA—the nucleus accumbens, Hp—the hippocampus, Ht—the hypothalamus, BS—the brain stem, SN—the substantia nigra, CB—the cerebellum, and RM—the remainder.

The CYP2D protein level was measured by Western blot analysis in the microsomes from selected brain structures of control and lurasidone-treated rats. Lurasidone exerted a significant effect on the CYP2D protein level in the brain. The observed changes in CYP2D protein level in most cases corresponded well with the changes in CYP2D activities. Lurasidone diminished the protein level in the frontal cortex (down to 84.5% of control) and in the hypothalamus (down to 66% of control) but enhanced the enzyme protein level in the striatum (up to 157% of control), nucleus accumbens (up to 131% of control, not significant), brain stem (up to 128% of control), substantia nigra (up to 131% of control), and in the remainder of the brain (up to 122% of control) (Figure 2). Lurasidone did not significantly affect the CYP2D4 protein level in the hippocampus, cerebellum, and in the liver (Table 1).

### 3.3. The Effect of Chronic Lurasidone Treatment on the CYP2D Gene Expression in the Rat Liver

**Figure 3:**
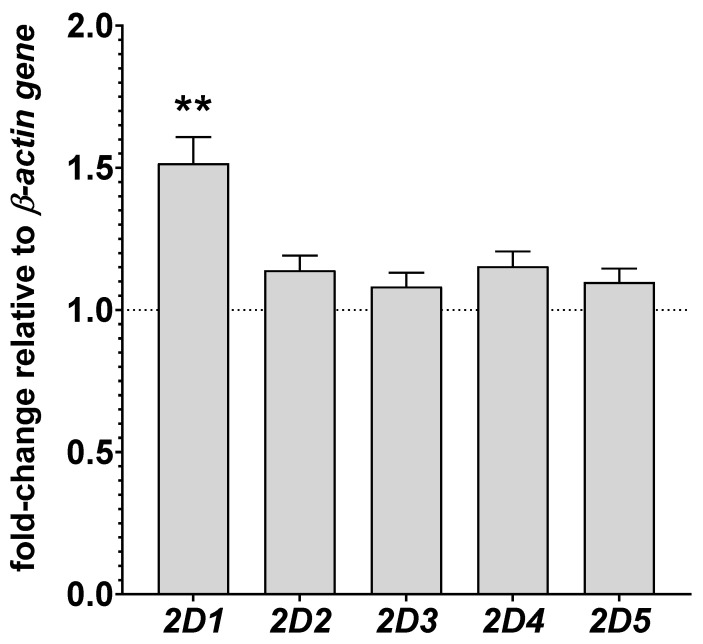
The influence of two-week treatment with lurasidone on the mRNA levels of the *CYP2D1*, *CYP2D2*, *CYP2D3*, *CYP2D4*, and *CYP2D5* genes in the liver. The results are expressed as the fold change compared to the ACTB housekeeping gene. The presented values are the mean fold change quantified by the comparative delta–delta Ct method for the control and lurasidone-treated rats (mean ± S.E.M. of 10 sample). Student’s *t*-test: ** *p* < 0.01 vs. control group.

The mRNA levels of CYP2D enzymes, i.e., *CYP2D1*, *CYP2D2*, *CYP2D3*, *CYP2D4*, and *CYP2D5* were measured in the liver. Lurasidone produced a significant increase in the mRNA level of *CYP2D1* gene, up to 151% of the control value (Figure 3). However, the neuroleptic did not significantly influence the expression of other examined *CYP2D* genes in the liver.

Considering the small size of brain structures (in particular, in the case of the nucleus accumbens and substantia nigra, we pooled three structures per one sample to measure the activity) and the fact that brain CYP2D may be induced at the posttranscriptional level, the effects of lurasidone on the *CYP2D* mRNA levels were not investigated in the brain in this study.

## 4. Discussion

The results of the present study show the impact of chronic administration of lurasidone on cytochrome P450 2D (CYP2D) activity and expression in the brain and liver. They indicate that lurasidone exerts organ- and brain structure-depended effects on the CYP2D enzyme.

The effect of the tested neuroleptic on brain CYP2D is different than that observed in the liver. In the liver, no changes in the activity and protein level of the total hepatic CYP2D enzyme were observed after prolonged administration of lurasidone, though the mRNA of CYP2D1, i.e., one of the six CYP2Ds expressed in the liver (CYP2D1, 2, 3, 4, 5, 18) was moderately increased. However, in the brain, where CYP2D4 is mainly expressed, the activity and protein level of CYP2D was altered in a cerebral structure-dependent way.

Our previous in vitro studies carried out on human liver microsomes and cDNA-expressed human CYP2D6 Supersomes showed a weak potency of lurasidone to inhibit the enzyme activity [17]. Thus, no direct interaction of lurasidone with human CYP2D6 protein (observed earlier) and lack of changes in the liver CYP2D activity after chronic neuroleptic treatment (observed in the present study) indicate that lurasidone has no potential for metabolic drug–drug interactions with CYP2D substrates during their biotransformation in the liver.

However, 14-day treatment with lurasidone influenced brain CYP2D, and the effect varied between cerebral regions. Lurasidone diminished the CYP2D activity and protein level in the frontal cortex but significantly enhanced the enzyme activity and protein level in the striatum, nucleus accumbens, brain stem, substantia nigra, and the remainder of the brain, which may modify its pharmacological effect. Reducing the CYP2D activity in the frontal cortex lurasidone may slow down the oxidative metabolism of neurosteroids (*via* 21-hydroxylation), thereby exerting beneficial effects on the symptoms of schizophrenia. It was shown that neurosteroids have a wide range of potential clinical applications for the treatment of schizophrenia [28,29] due to their ability to modulate GABA_A_ and NMDA receptors and neuroprotective properties [30,31].

In contrast, lurasidone increased the CYP2D activity and protein level in the striatum, substantia nigra, nucleus accumbens, brain stem, and remainder of the brain. Thus, lurasidone may increase the local synthesis of monoamonergic neurotransmitters catalyzed by CYP2D, i.e., tyramine hydroxylation to dopamine and 5-methoxytryptamine *O*-demethylation to serotonin [25,32]. The stimulation of CYP2D-mediated dopamine formation in the nigrostriatal pathway and serotonin formation in the brainstem may alleviate extrapyramidal symptoms of the neuroleptic [33,34].

The observation that lurasidone did not produce any changes in the expression and activity of liver CYP2D (in particular CYP2D4), while it did in the region-dependent manner in the brain (where CYP2D4 is the main CYP2D enzyme), implies that the neuroleptic exerted its effect on the brain enzyme via acting at monoaminergic receptors, and all the more so as similar effects on brain CYP2D in the frontal cortex and nigrostriatal pathway were observed for the atypical neuroleptics previously studied by us, namely, iloperidone and asenapine, which share with lurasidone similar receptor mechanisms in the brain [15,16]. These three atypical neuroleptics act at different types/subtypes of dopaminergic, serotonergic, or noradrenergic receptors (Table 2), which are heterogeneously distributed throughout the brain in different structures and on different types of neuronal and glial cells. They usually function through metabotropic receptors coupled negatively (e.g., D_2_, 5-HT_1A_) or positively (e.g., D_1_, 5-HT_2A_, 5-HT_6_, 5-HT_7_) via G-protein to adenylate cyclase or C-phospholipase and A_2_-phospholipase to evoke second-messenger intracellular signaling (reviewed by [35,36]). Because of differential regional distribution of those monoaminergic receptors and transcription factors in the brain, the regulation of a variety of biologically active proteins including cytochrome P450 enzymes is also region-dependent (discussed by [37,38]). For example, in the prefrontal and frontal cortex, D_1_ receptor dominates over D_2_ receptor compared to other brain areas, which may be a reason for different regulation of CYP2D4 in this brain area compared to the striatum or nucleus accumbens. In contrast, the mentioned atypical neuroleptics display different affinities for dopaminergic D_1_, D_3_, D_4_, 5-HT_6_, α_1_, and histaminergic H_1_ receptors (Table 2), which also may influence their effect on brain CYP2D4 activity/protein level.

Lurasidone is an atypical antipsychotic with a unique receptor-binding profile including antagonism at the dopamine D_2_ receptor (but not other dopaminergic receptors), serotonin 5-HT_2A_ and 5-HT_7_ receptor, and partial agonism at the 5-HT_1A_ receptor [39]. Recent studies by Fukuyama et al. [40] showed that subchronic lurasidone administration attenuated adenosine monophosphate-activated protein kinase (AMPK) and extracellular signal-regulated kinase (ERK) signaling in cultured astrocytes due to its action at 5-HT_7_ receptors. In other studies, the suppression of AMPK increased CCAAT/enhancer-binding protein (C/EBPβ) and pCREB expression in hepatoma cells [41], while overexpression of C/EBPβ enhanced mRNA levels of *CYP2D* in HepG2 cells [42]. It is also worth noting that chronic administration of lurasidone attenuated ERK signaling, which was related to the combination of long-term inhibition of D_2_, 5-HT_2A_, and 5-HT_7_ receptors and down-regulation of 5-HT_1A_ and 5-HT_7_ receptors [40]. The inhibition of ERK may contribute to an increase in the PPARγ activity and, in turn, to the elevation of CYP2D expression. Since PPARγ contains a mitogen-activated protein kinase (MAPK) site, the phosphorylation by ERK leads to the inhibition of PPARγ activity [43,44]. In addition, the CYP2D6 gene is positively regulated by PPARγ, as shown in vitro in neuroblastoma SH-SY5Y cells and in vivo in the cerebellum and liver of mice [45]. Thus, the observed increases in the CYP2D activity/protein in specific brain structures in our experiment may be due to the receptor action and intracellular molecular effects of lurasidone. Further molecular studies will show which receptor–transduction pathway is involved in the regulation of CYP2D in particular brain regions and whether the enzyme regulation proceeds at a transcriptional or posttranscriptional level.

## 5. Conclusions

In conclusion, lurasidone regulates brain (but not liver) CYP2D activity/protein level in a region-dependent manner, which is similar to that of other atypical neuroleptics (iloperidone and asenapine) as concerns frontal cortex (down-regulation) and nigrostriatal pathways (up-regulation), and may be of pharmacological significance. However, further molecular studies with selective receptor agonists are necessary to find out which individual monoaminergic receptors/signaling pathways and transcription factors are involved in the rat CYP2D4 and human CYP2D6 enzyme regulation in particular brain structures.

## Figures and Tables

**Table 1:** The CYP2D activity and protein level after lurasidone treatment in the brain structures and liver.

Tissue		CYP2D Activity (% of Control)	CYP2D Protein Level (% of Control)
**Brain structures**	**FCx**	77 ± 6.7 ↓ *	84.5 ± 4.8 ↓ *
	**St**	131 ± 15.3 ↑ **	157.2 ± 10.8 ↑ *
	**NA**	175.3 ± 42.9 ↑ *	131.8 ± 11.2
	**Hp**	88.9 ± 16.7	96.4 ± 16.4
	**Ht**	99.7 ± 10.4	66.9 ± 4.6 ↓ *
	**BS**	133.9 ± 17.4 ↑ *	128.9 ± 8.9 ↑ *
	**SN**	154.5 ± 35.9 ↑ *	131.5 ± 5.1 ↑ *
	**Cb**	98.7 ± 23.3	92.2 ± 12.4
	**RM**	115.9 ± 15.8 ↑ *	122.4 ± 11.1 ↑ *
**Liver**		106.1 ± 28.6	106.6 ± 6.7

↑, ↓ Increase or decrease, respectively. Student’s *t*-test: * *p* < 0.05 vs. control group; ** *p* < 0.01 vs. control group. FCx—the frontal cortex, St—the striatum, NA—the nucleus accumbens, Hp—the hippocampus, Ht—the hypothalamus, BS—the brain stem, SN—the substantia nigra, Cb—the cerebellum, and RM—the remainder.

**Table 2:** The effects of novel atypical neuroleptic drugs on the CYP2D activity and protein level in the liver and brain.

Drug	Liver	Brain Structures								
		FCx	St	NA	Hp	Ht	BS	SN	Cb	RM
Lurasidone (D_2_, 5-HT_2A_, 5-HT_7_, 5-HT_1A_, α_2C_)	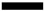	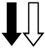	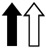	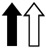	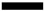	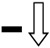	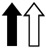	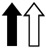	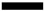	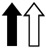
Iloperidone (D_2_, D_3_, 5-HT_2A_, α_1_, α_2_)	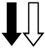	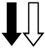	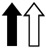	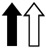	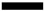	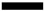	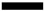	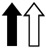	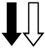	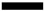
Asenapine (D_1_, D_2_, D_3_, D_4_, 5-HT_1A_, 5-HT_1B_, 5-HT_2A_, 5-HT_2B_, 5-HT_2C_, 5-HT_5A_, 5-HT_6_, 5-HT_7_, α_1_, α_2_, H_1_)	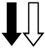	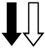	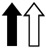	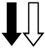	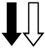	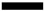	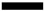	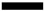	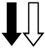	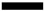

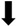
, 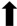
—increase or decrease in activity, respectively; 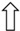, 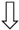
—increase or decrease in protein level, respectively; 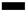
—no effect. Receptors placed in the brackets display K_i_ below 20 nM [11]. FCx—the frontal cortex, St—the striatum, NA—the nucleus accumbens, Hp—the hippocampus, Ht—the hypothalamus, BS—the brain stem, SN—the substantia nigra, Cb—the cerebellum, RM—the rest of brain.

## Data Availability

The data are contained within the article.
